# Understanding the clinical morbidity and mortality of fibrodysplasia ossificans progressiva: a systematic literature review

**DOI:** 10.1186/s13023-025-03763-8

**Published:** 2025-05-31

**Authors:** Hind Harrak, Susan Rhee, Amal Souttou, Sarah J. O’Meara, Caitlin Knox

**Affiliations:** Regeneron Pharmaceuticals, Inc., Tarrytown, NY USA

**Keywords:** Comorbidity, Epidemiology, Fibrodysplasia ossificans progressiva, Medication use, Mortality, Systematic literature review

## Abstract

**Background:**

Fibrodysplasia ossificans progressiva (FOP) is an autosomal dominant ultra-rare disease characterized by progressive episodic multi-focal heterotopic ossification of skeletal muscle, ligaments, tendons, and fascia. Comprehensive characterization and understanding of the natural disease history of FOP, including mortality, comorbidities, and medication use, is currently limited.

**Objective:**

This systematic review, which we believe is the first of its kind, aims to synthesize current knowledge on the morbidity, mortality, and medication use associated with FOP.

**Methods:**

A systematic literature review (SLR) was conducted utilizing various databases including PubMed, Embase, the Cochrane Central Register of Controlled Trials, the Trip database, and National Institute for Health and Care Excellence documents. The search was limited to studies involving humans, but was not restricted based on language. The studies used reported at least one of the following outcomes: mortality, comorbidity, and medication use; any clinical trials that were solely designed to evaluate a symptomatic treatment for FOP, such as flare-ups and pain, were excluded. Two independent reviewers reviewed and selected the included studies of the review, and extraction was done by one reviewer with cross-check performed by the other person. The Joanna Briggs Institute Critical Appraisal Checklist, specifically designed for studies reporting prevalence data, was used to assess the quality of studies. The SLR was registered on Prospero (CRD42022366914).

**Results:**

In total, 32 publications were selected for review. These studies included a wide range of participants age (0.1–78 years), study duration (1 day–33 years), and sample size (3–299 patients). Ten studies reported information on mortality, 26 studies reported on comorbidities, and 12 reported information on medication use. The three organ systems most affected by the conditions studied were, in order of severity, the cardiovascular (40–70%), skeletal (50–65%), and respiratory (20–42%).

**Conclusions:**

Although FOP is an ultra-rare disease, the available literature demonstrates that it is associated with excess morbidity and mortality. Our review synthesized all available published estimates of epidemiologic landscape of FOP and demonstrates the need for future work to better understand this rare disease.

**Supplementary Information:**

The online version contains supplementary material available at 10.1186/s13023-025-03763-8.

## Background

Fibrodysplasia ossificans progressiva (FOP) is an ultra-rare disease characterized by episodic soft tissue heterotopic ossification (HO) [1]. A bilateral great toe malformation and postnatal skeletal dysplasia are clinical hallmarks for FOP [2]. The genetic mutation causing FOP was first described in 2006 [3]; however, a clear description of what is very likely FOP appears in a case report in 1736 [4]. Since the discovery of heterozygous mutations of the activin A receptor, type I (*ACVR1*) gene in 2006 and the subsequent establishment of the causative pathway of FOP [3], molecular testing has been used to confirm clinical diagnosis. The global estimated prevalence of FOP varies between 0.36 and 1.36 per million people [5–7]. However, these numbers could be under-estimating the true prevalence of the disease due to difficulties in diagnosis, and limited population-based estimates of the disease.

Currently there is limited understanding of the natural history of rare diseases in general, and there remains a need for prospective, protocol-driven studies at the earliest stages of drug development [8]. Previous studies on FOP have predominantly been aimed at understanding the root cause of the disease, with a particular emphasis on identifying genetic variants and comprehending the triggers of flare-ups (episodes of soft tissue swelling, pain, reduced movement, stiffness and warmth) and HO. However, comprehensive data regarding the natural progression of the disease, including its associated morbidity and mortality rates, is notably lacking and is often based on isolated cases or anecdotes. While deafness and cardiorespiratory issues are generally accepted as common comorbidities within this population, the exact percentage of people affected by these conditions remains uncertain. Additionally, the presence and impact of other comorbidities on people with FOP are not clearly defined. To date, there are no population-based studies or systematic literature reviews (SLRs) that evaluate the existing data on the morbidity and mortality of FOP. This SLR synthesizes comprehensive data on both morbidity and mortality associated with FOP, providing a holistic view of the disease's impact. We believe this will be the first to systematically quantify the prevalence of comorbidities and mortality in people living with FOP, offering new insights into the clinical progression and long-term outcomes of the disease. Our findings aimed to fill a critical gap in the existing literature and provide support for a path forward for future research to better understanding this rare disease.

## Methods

This SLR was structured to provide a comprehensive evaluation of publicly available information on the distribution and determinates or epidemiology of FOP. Our systematic review process involved two independent reviewers (HH and XQ) who completed the study selection and reviewed the identified literature. Both reviewers independently screened the titles and abstracts of all identified studies for relevance. This was followed by a thorough review of the full text to extract a priori defined data and to categorize the type of study design. An initial data extraction process, completed in May 2023, was performed by one reviewer (HH) and was subsequently reviewed by a second independent reviewer (IM). Any disagreements were resolved by a third reviewer (CK). The literature search was extensive and robust, to ensure all available studies on morbidity and mortality among those living with FOP was captured.

### Performed searches

An initial comprehensive screening of PubMed (from inception to December 31, 2022), Embase (from 1974 to the second week of December 2022), and the Cochrane Central Register of Controlled Trials [9] (from inception to December 31, 2022), as well as the Trip database [10], which is a clinical search engine used to find research evidence using the Population, Intervention, Comparison, and Outcome (PICO) search format, the National Institute for Health and Care Excellence [11], and clinicaltrials.gov (without any time restrictions) was conducted. The PubMed search string was as follows: "Fibrodysplasia Ossificans Progressiva" OR "Progressive Myositis Ossificans" OR "Progressive Ossifying Myositis" OR "Myositis Ossificans Progressiva"; and the following Embase search string: 'fibrodysplasia ossificans progressiva'/exp OR 'ossifying myositis'/exp OR "fibrodysplasia ossificans" OR "hyperplasia, progressive facial" OR "muscle, ossifying myositis" OR "myopathy, osteoplastic" OR "myositis calcificans" OR "myositis ossificans" OR "myositis ossificans progressiva" OR "myositis, progressive ossifying" OR "neurogenic fibrodysplasia, ossifying" OR "neurogenic ossifying fibrodysplasia" OR "neurogenic ossifying myositis" OR "neurogenic osteoarthropathy" OR "neurogenic paraosteoarthropathy" OR "ossifying fibrodysplasia" OR "ossifying fibrodysplasia, neurogenic" OR "ossifying myositis, progressive" OR "osteoplastic myopathy" OR "para osteoarthropathy, neurogenic" OR "progressive myositis ossificans" OR "progressive ossifying myositis". No language restrictions were placed on any of the searches. Additionally, HH and XQ manually reviewed the reference lists of the selected studies to identify any additional publications that may not have been found through the keyword search.

An additional PubMed and Embase search for articles published between January 1, 2023 and June 28, 2024 was conducted to capture more recent publications by HH on June 28, 2024, using the same search string described above.

### Study selection

Studies were deemed eligible for inclusion if they met the following criteria: (a) the study population consisted of people diagnosed with FOP, inclusive of all FOP variants; and (b) the study reported on at least one of the outcomes of interest, namely mortality, comorbidity, or medication use within the population of people living with FOP. Studies were excluded if: (a) they were conducted in vitro, in vivo, or ex vivo; (b) they were case reports; (c) the results were published more than once, in which case only the most comprehensive study was retained; or (d) the reported results were from a clinical trial aiming to treat FOP. In cases where there was no English abstract available or the abstract was not sufficient to establish eligibility for inclusion, publications were shared with authors or colleagues based on the language(s) they master, along with instructions on inclusion/exclusion criteria. There were no studies published in non-English that were eligible to be included in this SLR.

### Outcomes of interest definitions

Mortality was defined as any reported death in the FOP population, including the number of deaths and the cause of death. Comorbidities were acute or chronic diseases or disorders, excluding HO and flare-ups which are direct manifestations of FOP. Medication use was defined as medication used to help control or treat comorbidities among those living with FOP.

### Quality assessment

The quality of each study was assessed using the Joanna Briggs Institute (JBI) Critical Appraisal Checklist for studies reporting prevalence data [12]. This quality-assessment tool evaluates population selection appropriateness, outcome-measure standardization, and analytical approach. The checklist is applied to each selected study individually.

### Data extraction

A standardized form was developed and used to ascertain study characteristics extraction and outcomes of interest as follows: (a) mortality rate, as reported within the original publication, and death counts (with cause of death when available); (b) comorbidities reported by the authors (with detailed measurements recorded if available); and (c) medication used to manage or treat comorbidities.

## Results

**Fig. 1:**
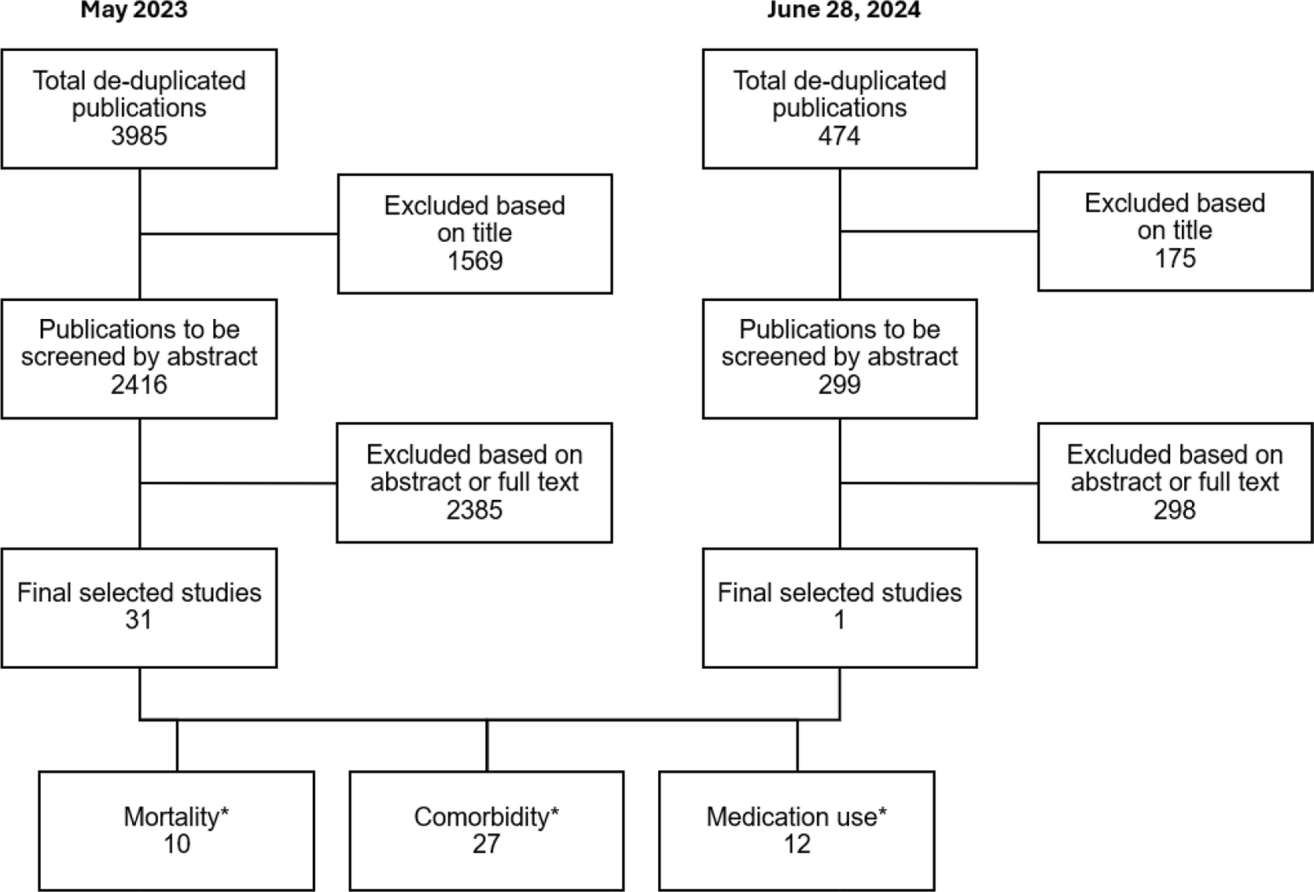
Study screening and selection flowchart. * These studies are not mutually exclusive, i.e., some studies reported across the three outcomes of interest

The initial databases search returned 3985 potential studies from 1968 through December 31, 2022, of which 2416 were selected for full review and 31 met the inclusion criteria (Fig. 1). The included studies were published between 1968 and 2022 and represented multiple countries worldwide. An additional search for more recent studies published between January 1, 2023, and June 28, 2024, identified approximately 107 and 367 publications from PubMed and Embase, respectively. Of an additional 299 studies identified, one, published in 2023, met the inclusion criteria and was added to this review. Therefore, the total number of publications included in this analysis was 32.

The design of the studies included varied, with the majority being observational (Table 1). Twelve were global studies, 11 were based in the USA only, three were in Asia, and six were in Europe. The total sample size varied from three to 299 patients; FOP diagnosis was self-reported in six studies, confirmed by a clinician through clinical presentation and medical history in 17, genetic testing in four, and by a combination of clinical manifestation and genetic testing in five. Patient demographics also varied by study (Table 1); follow-up periods ranged from 1 day to 33 years. Mortality data were reported in 10 of the 32 selected studies, but only one study determined the lifespan and causes of mortality in people living with FOP. Comorbidities data were extracted from 27 publications. Data on medication used by people living with FOP were available in 12 of the 32 studies (Table 1).

**Table 1:** Descriptive summary of studies

Study	Year	Location	Design	Patients, n	Age range/mean age/median age (years)	Setting	Follow-up	FOP diagnostic criteria	Mortality	Morbidity	Medication use
Botman et al.	2021	Netherlands	Observational—retrospective cohort (chart review)	7	7–57	Outpatient	6–18 years	Physician—clinical manifestation		X	
Connor et al.	1981	UK	Interventional—single arm	21	6–70	Outpatient	Unknown	Physician—clinical manifestation		X	
Connor et al.	1982	UK	Observational—descriptive cohort involving surveys and record review	34	5–71	Outpatient	Unknown	Physician—clinical manifestation	X	X	X
Forrest et al.	2022	USA	Observational—case series	4	18–27	Inpatient	N/A	Genetic testing		X	
Fujihara et al.	2022	Japan	Interventional—single arm	3	18–43	Outpatient	20 years	Unclear—likely by physician			X
Glaser et al.	1998	FOP group: Global Control group/non-FOP: USA	Observational—retrospective Cohort study (survey)	112	2–75	Outpatient	N/A	Self-reported	X	X	
Gupta et al.	2018	Global	Observational—cross-sectional (survey)	207	3–75	N/A	N/A	Self-reported		X	X
Janoff et al.	1996	USA	Observational—retrospective cohort (chart review)	107	6–47	Outpatient	17 years	Physician—clinical manifestation	X	X	
Kaplan et al.	2010	Global	Observational—cross-sectional (medical records)	60 deaths and 371 living	3–60	Outpatient	33 years	Physician—clinical manifestation	X		
Keen et al.	2017	UK	Observational (telephone survey)	17	Unknown	N/A	N/A	Physician—clinical manifestation			X
Kilmartin et al.	2014	USA	Observational—retrospective chart review	30	5–64	Outpatient	N/A	Physician—clinical manifestation		X	
Kitterman et al.	2012	Global	Observational (survey)	168	1.5–60	N/A	N/A	Self-reported		X	X
Kou et al.	2020	Global	Observational—prospective descriptive cohort (survey)	106	4–56	Outpatient	1 year	Physician, genetic testing confirmation		X	X
Kou et al.	2022	Global	Observational—retrospective and prospective cohort	159	1.4–76	Retrospective: N/A Prospective: Outpatient	2 years	Physician—clinical manifestation		X	X
Kussmaul et al.	1998	USA	Observational—cross-sectional	25	5–55	Outpatient	N/A	Physician—clinical manifestation		X	
Lanchoney et al.	1995	USA	Observational (survey)	23	1.25–21	N/A	N/A	Self-reported			X
Levy et al.	1999	USA	Part I: Observational (survey) Part II: Observational—descriptive cohort	54 7	Part I: 1–60 Part II: 11–43	Survey: N/A Cohort: Outpatient	N/A N/A	Part I: Self reported Part II: Self-reported		X	
Lindborg et al.	2023	Global	Observational—case series	31	5–57	Outpatient		Physician and genetic testing		X	
Ludman et al.	1968	UK	Observational—case series	3	15–38	Outpatient	N/A	Physician—clinical manifestation		X	
Morales-Piga et al.	2012	Spain	Observational—retrospective cohort	24	4–53	Partial outpatient	N/A	Physician—clinical manifestation	X	X	
Moriatis et al.	1997	USA	Observational—retrospective cohort (chart review)	74	1–49	Outpatient	17 years	Physician—clinical manifestation	X	X	X
Muglu et al.	2012	USA and UK	Observational—case series	4	22–27	Inpatient	N/A	Physician and genetic testing		X	
Peng et al.	2019	Global	Observational—retrospective cohort	99	18–74	N/A	2.5 years	Physician—clinical manifestation		X	
Peng et al.	2021	USA	Observational—prospective cohort	17	7–61	Outpatient	1 month	Physician and genetic testing		X	X
Pignolo et al.	2022	Global	Observational—prospective cohort	114	4–56	Outpatient	3 years	Genetic testing	X	X	X
Pignolo et al.	2021 (NHS)	Global	Observational—prospective cohort	114	Unknown	Outpatient	3 years	Genetic testing		X	
Pignolo et al.	2020	Global	Observational (survey)	299	0.1–78	N/A	N/A	Self-reported		X	
Pignolo et al.	2021 (prevalence)	USA	Observational—cross-sectional	373	Unknown	Outpatient	N/A	Physician—clinical manifestation	X		
Shah et al.	1994	USA	Observational—retrospective cohort (chart review)	40	1–38	Outpatient	16 years	Physician—clinical manifestation		X	
She et al.	2020	China	Observational—retrospective cohort (chart review and survey)	65	Unknown	Outpatient	10 years	Physician and genetic testing	X	X	
Wentworth et al.	2018	USA	Observational—case series (chart review)	3	27–53	N/A	N/A	Physician—clinical manifestation	X	X	
Zhang et al.	2020	China	Observational—retrospective cohort (chart review)	26	Unknown	Inpatient	12 years	Physician or genetic testing (10 patients)		X	X

*FOP* fibrodysplasia ossificans progressiva, *N/A* not applicable, *NHS* National Health Service

### Mortality

Ten of the 32 publications reported the number of deaths within the study cohorts [5, 6, 13–20]. However, none of the studies calculated annual mortality rates or contextualized the number of deaths observed. She et al. has reported a cumulative cohort mortality rate of 13.8% over the course of 10 years [13]. In 2006, Kaplan et al. reported 60 deaths over 33 years (between 1973 and 2006) and 371 people living with FOP in 2006; no mortality rate was reported and the reported median age at time of death was 40 years old. The Kaplan–Meier survival analysis conducted by the authors showed a median life expectation of 56 years (95% confidence interval: 51–60 years) [16].

In seven of the 10 publications which reported deaths, the cause of death was reported. The most frequently reported causes of death, as determined by the investigators’ medical records review, were cardiovascular or cardiorespiratory issues, pneumonia, and trauma resulting from a fall.

Wentworth et al. [14] was the only study that provided complete or partial autopsy results from a case series. Within the case series, one patient’s cause of death was identified as head trauma following a fall, leading to intracranial hemorrhage. The other two patients died due to respiratory failure and thoracic insufficiency.

### Comorbidity

In our SLR, data on FOP comorbidity were extracted from 27 of the included studies, encompassing 73 distinct diseases and disorders. The three organ systems most affected by the conditions studied were, in order of severity, the cardiovascular (40–70%), skeletal (50–65%), and respiratory (20–42%) (Table 2). The majority of the conditions and disorders (55 out of 73) were reported from a single article.

**Table 2:** Ten most reported morbidities and conditions

Organ system and assistive devices	Morbidity	Studies reporting condition, n	Patients reported with condition, %
Ear, nose and throat	Deafness	9	34
	Earaches or drainage	1	25
Neurology	Chronic pain	5	21
	Chronic migraine	2	27
	Poor sleep quality	1	21
	Increased sleep latency	1	18
Lung system	Dyspnea	2	18
	Lung disease	6	21
Assistive devices	Wheelchair use	5	54

Hearing loss as a comorbidity of FOP was reported in eight of the 26 studies [5, 13, 15, 19, 21–24]. The prevalence of hearing loss among individuals with FOP varied, with a reported frequency of 5% in a population under 18 years of age [13], and 52% among survey respondents with an average age of 25 years [21]. When specified, the majority of cases were mild, either self-reported by patients or measured as a hearing loss ranging from 25 to 40 decibels [21]. Earaches and ear drainage issues were also prevalent, affecting 25% of the 195 patients surveyed in one study [24].

Within the category of cardiovascular comorbidities, conduction abnormality emerged as the predominant described disorder, with the majority of these being identified from electrocardiogram readings. Just under half of these readings indicated some form of abnormality [25, 26]. However, the authors of these studies did not provide reports on clinical cardiovascular outcomes that may result due to the observed conduction abnormality.

Dermatological manifestations were predominantly reported by patient surveys, with rashes and skin ulcers accounting for 18% and 13% of comorbidities in this category, respectively [24]. An examination of 116 surveyed female patients with FOP revealed the presence of nine distinct endocrine disorders, with menstrual irregularities being the most common, affecting 13% of the female participants out of 116 [24]. Gastrointestinal disorders were also noted in two studies [19, 24]. In one study 38% of respondents reported a locked jaw, followed by abdominal pain (15%), heartburn (12%), and nausea (12%) [19]. Despite the lack of data on infection rates in the 32 studies included in this review, pneumonia and lung infections were documented in 16% [19, 27] and 15% [24, 25] of patients, respectively [19, 24, 25, 27]. The publications lacked details on the severity of infections and did not provide any information on non-respiratory infections.

Of the 32 studies reviewed, pulmonary function disorders were described frequently. Lung disease, often un-defined or broadly categorized as a ventilatory dysfunction, was documented in six studies [14, 19, 23, 27–29]. Pignolo et al. [19] identified restricted chest expansion as the predominant respiratory disorder, reported by 42% of the patients surveyed. Neurological disorders were primarily represented by chronic pain [14, 19, 24, 30, 31], sleep disorders [24], and depression [19].

Outcomes of pregnancy were documented in two case series, encompassing a total of seven observed pregnancy events [32, 33]. Of these, 30% culminated in spontaneous abortion. There were five instances of live premature births; notably, two of these five neonates were diagnosed with FOP after birth. They presented with big toe malformations, and their diagnosis was later confirmed with genetic testing [32, 33].

Lastly, skeletal disorders were noted in eight out of the 32 studies included. Falls and fractures were prevalent, affecting roughly 50% of the 260 patients with FOP collectively included in three of the eight studies reviewed [15, 19, 20]. A case series analyzing 31 patients who sustained fractures of the normotopic skeleton found that when treated non-operatively, healing occurred 97% of the time, with few flare-ups and HO, and preservation of mobility [34]. Scoliosis was identified in 65% of the patients assessed in two studies [27, 35], while abnormal growth or knee bone abnormality was observed in 39% of the 66 pediatric patients in one study [36]. Furthermore, wheelchair usage was reported in about half of the patients across five studies [14, 18, 27, 28, 37].

### Medication and vaccine use

We extracted data on medication use among people living with FOP from 12 publications and found that medication was primarily used for managing chronic pain and inflammation (Table 3). Non-steroidal anti-inflammatory drugs (NSAIDs) and their chronic use were highlighted in three studies [19, 30, 37]. Opioid usage was also frequently discussed in three publications. Corticosteroids (with an unspecified administration route) and other anti-inflammatory preparations were mentioned in three studies [15, 19, 23].

**Table 3:** Reported drug use among patients with FOP by medication or medication class

n	Connor et al., 1982 (N = 34)	Kitterman et al., 2012 (N1 = 44—headache; N2 = 17—neuropathic pain)	Kou et al., 2020 (N = 106)	Moriatis et al., 1997 (N = 74)	Peng et al., 2021 (N = 17)	Zhang et al., 2020 (N = 26)	Forrest et al., 2022 (N = 3)	Kou et al., 2022 (N = 159)	Pignolo et al., 2022 (N = 114)	Gupta et al., 2018	Fujihara et al., 2022	Lanchoney et al., 1995
Corticosteroids	14								29			
Disodium etidronate	13											
NSAIDs	2	n1 = 28; n2 = 9		6	8				41			
Narcotics		n1 = 4; n2 = 3			9				5			
Gabapentin/pregabalin		n2 = 4			2							
Anti-migraine medication		n1 = 3										
Paracetamol			33						13			
Antihistamines			18						9			
Warfarin				2								
Glucocorticoid + NSAID						6						
Glucocorticoid + NSAID + cyclosporine						3						
Glucocorticoid + NSAID + methotrexate						8						
Nifedipine							1					
Labetalol							1					
SARS-CoV-2 vaccine								15				
Leukotriene receptor antagonist									21			
Proton pump inhibitor									13			
Anti-inflammatory preparation*									10			
Vitamins (multi or single)									8			
Anti-rheumatic products *									6			
Calcium									5			
Potassium citrate										3		
Antibiotics											1	
DTP vaccine												22

*DTP* diphtheria, tetanus, and pertussis, *FOP* fibrodysplasia ossificans progressiva, *NSAID* non-steroidal anti-inflammatory drug

Blank cells mean that the specific drug was not reported in the study

^*^These categories are reported as they were in their respective studies, no detail was available on the specific drugs included

Vaccination among people living with FOP was also described in a few publications. A study by Kou et al. [38] noted that SARS-CoV-2 vaccines were generally well tolerated within the study population; 15 of 32 patients received the vaccine, predominantly via the intramuscular route (12 out of 15), one patient received the vaccine via subcutaneous injection, one patient received the first dose with a subcutaneous injection and the second was intramuscular, and the third did not report the injection site. No HO formation was reported post injection. Another study looked at vaccinations with the diphtheria, tetanus, and pertussis vaccine, which included data from 22 people living with FOP [39], with six patients developing HO at the injection site within 6 weeks.

### Quality assessment of selected studies

Utilizing the JBI Critical Appraisal Checklist for studies reporting prevalence data, significant issues were identified in the majority of items included in our SLR. Specifically, in 23 out of the 32 studies, either the sample frame and the methodologies employed for participant sampling were not appropriate, or there was not a clear enough description given to be able to review the appropriateness of the methods used. Moreover, outcomes of interest were not explicitly defined in the methods section of the studies, leading to uncertainty on how the outcome of interest was measured and whether the methods used were consistent and robust across all study participants.

## Discussion

Comprehensive data on the natural history of FOP is severely limited, which limits understanding of the disease. Our aim was to understand the current research surrounding the morbidity and mortality of FOP, outside of the traditional focus of FOP clinical trial research, which is usually HO formation. The SLR identified 32 studies reporting on the morbidity, medication use, and mortality of people living with FOP. Examined collectively, the prospective and retrospective, observational and investigational data reported from these studies will help explain the global epidemiology of FOP, as well as help guide resources, research, and interventions for this highly disabling genetic condition.

This SLR is the first to capture a holistic description of the FOP community and the challenges it faces using currently available published literature. Our findings suggest that there is a dearth of information on the concurrent health issues of people living with FOP and highlights the need for further research in this area. From the studies included in our analysis, we identified that the leading causes of death in people living with FOP are cardiovascular or cardiorespiratory issues, pneumonia, and trauma resulting from falls. These partially align with the most reported comorbidities in this community, which were cardiovascular, neurological, and pulmonary disorders. Chronic pain, which is categorized as a neurological disorder, was reported in several studies examining chronic use of NSAIDs. Interestingly, despite the widespread use of NSAIDs in this community, no studies to date have specifically evaluated their impact on the gastrointestinal system.

Since the early definition of FOP, and the description of the causative pathway, case reports still constitute the vast majority of publications reporting on people living with FOP and their characteristics. The anecdotal nature of this type of reporting limits the generalizability of findings, identification of disease patterns, and safety markers. The main sources of information about people living with FOP, as identified in this SLR were surveys and questionnaires completed by the patients themselves. Furthermore, studies that used electronic medical records, identified patients through their treating physician or were followed at the clinic where the study took place. Thus, the current population-based estimates have primarily relied on patient-reported data and direct clinical observations. This approach restricts patient selection to those known by the authors or identified through specific clinical settings, introducing the potential for selection bias, recall bias, and limits the generalizability of the results to the broader population of FOP.

An incredible effort has been undertaken by the International FOP Association (IFOPA) in organizing FOP patient associations across the world, which identify people living with FOP through their membership structure and collect data prospectively through patient-reported registries. These initiatives have helped support research efforts and advances in disease knowledge; however, there are still major gaps in our understanding. High-quality data from more geographical regions are necessary to understand global patterns and the ways that diverse pathophysiological etiologies may affect patterns of FOP.

Our review of medication use has also highlighted a high frequency and chronic usage of NSAIDs, along with other pain medication, further emphasizing the burden of chronic pain management in this population.

Through our unique research, we have identified, collected, and summarized all currently available published data on clinical outcomes in the FOP community, and have highlighted many unanswered questions. Although we were able to include 32 publications, we still cannot derive an accurate mortality rate or accurate prevalence rates for common comorbidities and other clinically relevant outcomes observed among people living with FOP due to the limited contextualization provided in the studies of both the selected participants and outcomes measures. Pregnancy outcomes are also one of the many examples where data are limited to a handful of cases, which results in an inability to assess outcomes and generalize findings to the overall population.

Unfortunately, it was difficult to pool the available data to be able to explore and identify any trends in the published literature, as much of the collected information originates from the same pool of people living with FOP, most clinical characteristics were only described once, and there was a lack of clear outcomes definitions. The epidemiological landscape of FOP therefore remains inadequately delineated in existing literature. Furthermore, prevalence data for the disease is notably sparse for North America and Europe, with a complete absence of FOP prevalence estimates in Asia and Africa. The paucity of detailed descriptions of patient characteristics and geographical distribution data is a significant hurdle for the design of future studies aimed at minimizing selection biases. These limitations, which were seldom addressed in the studies identified, further impede their generalizability. The lack of accurate baseline population estimates may complicate the determination of the appropriate sample size for a study of people living with FOP. Another common limitation observed in studies that employ surveys for data collection is the absence of standardized and reliable data collection methodologies. Unfortunately, the included studies did not explicitly define the outcomes of interest in the methods section of the publications, leading to a degree of ambiguity in their representation in the survey questions.

The limited ability to identify people living with FOP through secondary data, has limited the study design choices made so far. Although multiple initiatives have been launched to better our understanding of FOP and its progression, there is still a large gap in our knowledge about this disease.

## Conclusions

The range of morbidity and mortality for people living with FOP varied widely across countries and studies, reflecting varying study designs and levels of knowledge of the natural history of this disease. Although FOP is a rare disease, the available literature demonstrates that there is excess morbidity and mortality associated with it. Our review evaluated all available published estimates of the epidemiologic landscape of FOP and demonstrated the need for future work to better understand this rare disease. The anecdotal nature of the findings limits the generalizability and reliability of the data. Additionally, there is a lack of standardized outcome definitions, making it difficult to draw consistent conclusions. The uncertain representativeness of the FOP community in the studies reviewed raises questions about the applicability of the findings to the broader population of individuals living with FOP. In light of the findings from our study, there is a clear need for more precise and standardized methodologies in future clinical research. Specifically, the standardization of comorbidity definitions is crucial to enhance the comparability and reliability of research outcomes, ensuring consistency across studies and facilitating more accurate meta-analyses. This could be supported by the utilization of a global disease registry, rather than one-off surveys. A global disease registry could significantly elevate the quality of data, consistency of data, and ability to follow patients longitudinally, as well as improve the range of information collected. This approach will enable the collection of more comprehensive and high-quality data, leading to more robust and generalizable findings. By implementing these methodological enhancements, future research can achieve greater precision and reliability, ultimately advancing our understanding and management of FOP.

## Supplementary Information

Supplementary material 1

## Data Availability

Qualified researchers may request access to study documents (including the clinical study report, study protocol with any amendments, blank case report form, and statistical analysis plan) that support the methods and findings reported in this manuscript. Individual anonymized participant data will be considered for sharing once the product and indication has been approved by major health authorities (eg, FDA, EMA, PMDA, etc.), if there is legal authority to share the data and there is not a reasonable likelihood of participant re-identification. Submit requests to https://vivli.org/.

## Abbreviations

IFOPA: International FOP association
FOP: Fibrodysplasia ossificans progressiva
HO: Heterotopic ossification
ICD: International Classification of Diseases
JBI: Joanna Briggs Institute
NSAID: Non-steroidal anti-inflammatory drug
SLR: Systematic literature review
