# Supplementary material for: Understanding the clinical morbidity and mortality of fibrodysplasia ossificans progressiva: a systematic literature review
Source: Orphanet J Rare Dis. 2025 May 31;20:262. doi: 10.1186/s13023-025-03763-8 (PMC12125817; doi:10.1186/s13023-025-03763-8)
Supplement: Supplementary file 1 — Supplementary material 1 [file 13023_2025_3763_MOESM1_ESM.docx]

# APPENDIX: PubMed search string

"Fibrodysplasia Ossificans Progressiva" OR "Progressive Myositis Ossificans" OR "Progressive Ossifying Myositis" OR "Myositis Ossificans Progressiva"; and the following Embase search string: 'fibrodysplasia ossificans progressiva'/exp OR 'ossifying myositis'/exp OR "fibrodysplasia ossificans" OR "hyperplasia, progressive facial" OR "muscle, ossifying myositis" OR "myopathy, osteoplastic" OR "myositis calcificans" OR "myositis ossificans" OR "myositis ossificans progressiva" OR "myositis, progressive ossifying" OR "neurogenic fibrodysplasia, ossifying" OR "neurogenic ossifying fibrodysplasia" OR "neurogenic ossifying myositis" OR "neurogenic osteoarthropathy" OR "neurogenic paraosteoarthropathy" OR "ossifying fibrodysplasia" OR "ossifying fibrodysplasia, neurogenic" OR "ossifying myositis, progressive" OR "osteoplastic myopathy" OR "para osteoarthropathy, neurogenic" OR "progressive myositis ossificans" OR "progressive ossifying myositis".
